# Animal cultures: how we've only seen the tip of the iceberg

**DOI:** 10.1017/ehs.2019.1

**Published:** 2019-05-23

**Authors:** Caroline Schuppli, Carel P. van Schaik

**Affiliations:** Department of Anthropology, University of Zürich, Winterthurerstrasse 190, 8057 Zürich, Switzerland

**Keywords:** culture, social learning, method of exclusion, orangutans, animal cultural repertoires

## Abstract

For humans we implicitly assume that the way we do things is the product of social learning and thus cultural. For animals, this conclusion requires proof. Here, we first review the most commonly used procedure for documenting animal culture: the method of exclusion, which charts geographic behavioral variation between populations as evidence for culture. Using published data, we show that, whereas it is an adequate proof of principle, the method of exclusion has major deficiencies when capturing cultural diversity and complexity. Therefore, we propose a new method, namely the direct counting of socially learned skills, which we apply to previously collected data on wild orangutans. This method reveals a far greater cultural repertoire among orangutans, and a different distribution of cultural elements among behavioral domains than found by the method of exclusion, as well as clear ecological correlates for most cultural elements. The widespread occurrence of social learning ability throughout the animal kingdom suggests that these conclusions also apply to many other species. Culture is most likely more widespread and pervasive than commonly thought and an important avenue to local adaptation. The complex and normative dimensions of culture seem unique to our species, but were most likely built upon a very broad, pre-existing cultural capacity that we inherited from our ancestors.

## Graphical abstract

**Figure:**
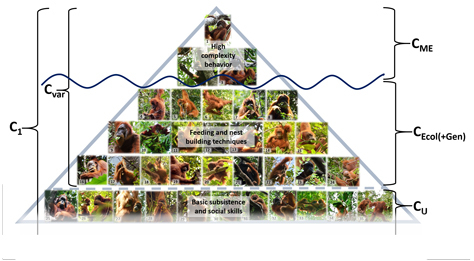

Social media summary: A new way to assess animal cultures suggests that culture is far more widespread and pervasive than commonly thought.

## Introduction

For a long time, culture was seen as an exclusively, and thus defining, human feature (Tylor, 1871). However, when evidence for culture-like variation was also found in a variety of non-human animal species, it became apparent that culture is part of a phylogenetic continuum rather than a uniquely human trait (Whiten, 2012). A clear definition of the phenomenon, universally agreed upon by different research fields, is called for, because the way we define culture has significant implications for where we will find it.

The oldest explicit definition by a cultural anthropologist regards culture as ‘that complex whole which includes knowledge, beliefs, arts, morals, law, custom, and any other capabilities and habits acquired by man as a member of society’ (Tylor, 1871). This human-oriented definition stresses that virtually everything we do, know or believe, as well as our institutions and technology, are a product of social learning and thus cultural (Braidwood, 1975). Notably, although the definition includes normative elements as products of culture, it does not claim that all culture is normative. It also leaves implicit how ‘complex’ culture has to be to qualify as such. In order to examine how human culture arose, biologists adapted this definition to non-human animals by describing culture as ‘all behaviors and knowledge that are acquired and passed on within and between generations through social learning’ (Boyd and Richerson, 1985). Hereafter, we refer to this as the minimal definition of culture.

Both definitions recognize that the precondition for culture is a demonstrated reliance on social learning, i.e. learning through observing, associating with or interacting with other individuals or their products (Heyes, 2012). However, whereas the minimal definition of culture as all socially learned skills and knowledge (at some point expressed in behavior) is still widely used by researchers studying culture in animals (Fragaszy and Perry, 2003; van Schaik, 2010; Whiten, 2017), some have argued that culture must be more than the product of social learning.

Thus, it has been proposed that socially learned behaviors can only qualify as cultural if they are normative, i.e. that an individual's deviant behaviors are sanctioned by its social group (Hill, 2009; McGrew, 2004). Others have argued that culture must be subject to cumulative change over time (Hill, 2009; Levinson, 2006; Tomasello *et al.*, 1993), and/or transmitted by high-fidelity forms of social learning such as imitation or teaching (Galef, 1992). Effectively, this means that only behaviors exceeding a minimum level of complexity qualify as cultural. Yet others have required transmission across multiple generations as an essential criterion (sometimes also referred to as traditions; Perry, 2009a, b; Whiten and van Schaik, 2007). Finally, it has been suggested that culture requires socially learned variants in a variety of behavioral domains (Whiten and van Schaik, 2007).

In short, all definitions agree that skills and knowledge that are learned through a form of social learning are the quintessence of culture, irrespective of the actual social learning mechanism used. The more demanding definitions appear to reflect an attempt to distinguish the cultures of different taxa, especially humans from animals, although many habits we naturally call culture in humans are neither normative nor complex or diverse. Yet if we see culture as an overarching concept, it allows us to see normativity, complexity, stability or diversity as aspects of culture, each with a phylogenetic distribution. Thus, the minimal culture definition (Boyd and Richerson, 1985) allows comparisons across species and allows us to find out about the evolutionary roots of a trait in which our species undeniably outranges all others. This approach allows us to study culture from the bottom up rather than assuming that the human case represents the gold standard. Following the minimal definition of culture, we do not differentiate between different levels of social learning, because most skills and knowledge, often in humans as well, can be transferred through simple forms of social learning (e.g. enhancement or socially induced practice) and do not require high-fidelity forms of social learning (e.g. imitation or teaching; van Schaik *et al.*, 2017). Accordingly, most behaviors that we will here consider as socially learned and thus cultural are within the innovative reach of an individual and not culture-dependent *per se* (Tennie *et al.*, 2009).

## How to Measure Culture?

When the minimal culture definition is applied to non-human animals, a significant problem arises. It is virtually impossible to demonstrate social transmission in nature, and where this was done unambiguously via field experiments, it was not clear whether the experimentally transmitted behaviors would persist over time (Reader and Biro, 2010; Whiten and Mesoudi, 2008). Thus, researchers have come to rely mainly on another approach, where they focus on one of the products of culture, namely geographic variation, to prove its presence.

If behaviors are acquired through social learning rather than invented independently, they are more likely to show geographic variation between populations because the underlying innovations are bound to be made only sporadically (Galef, 1976; Nishida, 1987). In humans, such contrasts between societies are automatically assumed to be the result of social transmission. However, for animals two alternative, non-exclusive explanations need to be excluded (McGrew and Tutin, 1978): (1) individually plastic responses to ecological differences between populations; and (2) population differences in genetic predispositions. This line of argumentation laid the foundation of the currently most commonly used tool to detect the presence of animal culture, namely the method of exclusion (MoE, also frequently referred to as the ethnographic method; Whiten *et al.*, 1999) in which we classify a behavior as cultural if we can demonstrate a high prevalence in some populations but its absence in others, but also can reasonably rule out genetic or ecological factors as causes of this behavioral difference (Galef, 1976; Nishida, 1987).

Ruling out ecological effects underlying behavioral variation is difficult, and ruling out genetic effects is next to impossible. Yet where it can be done convincingly (e.g. Krützen *et al.*, 2011; Lycett *et al.*, 2009, 2011, but also Langergraber *et al.*, 2010; Langergraber and Vigilant, 2011), we can identify cases where it is highly plausible that the observed variation is indeed the result of cultural transmission, especially when backed up by data on the social transmission process. The MoE is therefore a very useful proof of principle (van Schaik, 2010). Since for many species detailed data on individual behavior acquisition is lacking, the MoE has been used to suggest the presence of culture in species known to be capable of social learning in captivity. Accordingly, the MoE has revealed cultural variation in various primate, cetacean and bird species (Catchpole and Slater, 2003; Hohmann and Fruth, 2003; Krutzen *et al.*, 2005; Ottoni and Izar, 2008; Perry *et al.*, 2003; Riebel *et al.*, 2015; Robbins *et al.*, 2016; Santorelli *et al.*, 2011; van Schaik *et al.*, 2003a; Whitehead and Rendell, 2014; Whiten *et al.*, 1999).

Although intended as a tool to detect the presence of culture (Galef, 1976; Nishida, 1987), the criteria of the MoE are now so ingrained that this operationalization has, to all practical purposes, reached the status of the definition of animal culture: cultural status is only assigned to behaviors that show geographic variation between populations (van Schaik, 2010). In other words, a ‘cultural behavior is one that is transmitted repeatedly through social or observational learning to become a population-level characteristic’ (Whiten *et al.*, 1999). This perspective entails that culture is not an individual-level, but rather a population-level trait (see below).

For a variety of species, the MoE has been used to describe and compare cultural repertoire sizes (Robbins *et al.*, 2016; Santorelli *et al.*, 2011; van Schaik *et al.*, 2003a; Whiten *et al.*, 1999). However, we can list several shortcomings of the MoE (van Schaik *et al.*, 2009) which conspire to produce biased and highly restrictive estimates of cultural repertoires.

First, social learning does not necessarily result in between-group heterogeneity. Identical ecological conditions are likely to bring about the same behavioral innovations in separate social units. Cultural transmission driven by social learning will thus inevitably produce similarities in behavioral repertoires between them, just as evolution driven by natural selection will often come up with the same genetic adaptations in similar environments. Whereas the latter is a widely acknowledged phenomenon, namely convergent evolution, the possibility of convergent culture is not yet widely acknowledged.

**Figure 1.**
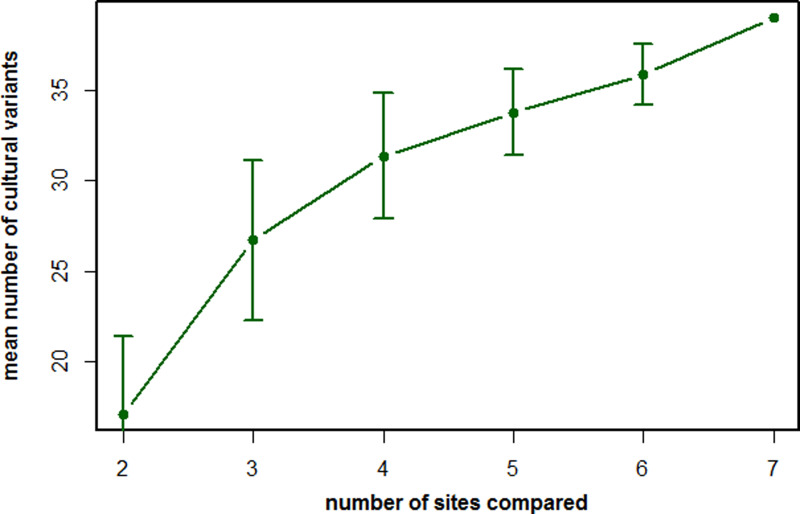
The number of recorded cultural variants as a function of populations being compared by the method of exclusion (MoE). The mean number of likely cultural variants described according to the criteria of the MoE by comparing the behavioral repertoires of an increasing number of chimpanzee populations. Based on data from Whiten *et al.* (1999).

Second, the MoE ignores all behavioral variants that covary with ecological factors, even if the behaviors are in fact socially learned and thus cultural (van Schaik *et al.*, 2009). Social learning will often produce behaviors that are adapted to a population's ecological conditions and help individuals to exploit natural resources (Byrne *et al.*, 2004). Thus naturally, a substantial part of a species’ cultural repertoire should indeed be linked to the local environment (Figure 1; Koops *et al.*, 2014; Laland and Janik, 2006). Human culture exemplifies that ecological differences drastically shape cultural behavior. Ecology may make it impossible for certain socially learned behaviors to appear in some regions, but their presence elsewhere still reflects cultural processes.

Third, because the MoE excludes the majority of cultural variation with ecological components, it is heavily biased in the kinds of cultural variation it captures (see below). Whereas it detects disproportionately more behaviors of the social domain and complex dietary inventions such as tool use, it underestimates culturally transmitted basic subsistence skills. However, it is highly unlikely that the mechanisms of social learning are exclusively deployed for the acquisition of complex innovations; routine subsistence skills are almost certainly acquired in the same way.

Fourth, the MoE treats genetic differences underlying behavioral variation between populations as a deal breaker for culture. Genetic differences indeed often correlate with behavioral differences, which suggests that genetic differences may play a role in creating behavioral differences (Langergraber *et al.*, 2010). Yet, in practice, genetic components do not rule out the presence of social learning (Laland and Janik, 2006; Krützen *et al.*, 2011). As with any complex phenotypic traits in nature, it is unlikely that behavioral repertoires can be strictly divided into innate and learned components. In particular, higher forms of social learning (Van Schaik, 2016; e.g. imitation, emulation and intentional teaching), which are based on a motivation to closely attend to the action of conspecifics, are likely to entail an element of genetically anchored social interest (Schuppli and van Schaik, 2019). According to the minimal definition of culture, behaviors based on genetic predispositions but expressed through socially mediated learning are to be considered as cultural.

Fifth, and perhaps most importantly, the number of cultural variants detected by the MoE is highly dependent on the number of populations we compare. Whereas a behavior X may well be shared between populations A and B, it may not be shared with population C. If we compare only populations A and B, behavior X will not be considered to be cultural, because it is found in all of the populations compared. If we then add population C to this comparison, behavior X will suddenly count as cultural because it is not universally shared among all compared populations. This inevitably leads to an underestimation of cultural repertoires.

To illustrate this last point, let us turn to the most extensive list of cultural elements for any animal species published to date, namely the chimpanzees (Whiten *et al.*, 1999). By comparing the behavioral repertoires of seven chimpanzee populations, a total of 39 behavioral variants were recognized, mainly composed of complex feeding skills (i.e. feeding techniques that contain multiple steps of processing, for example tool use) as well as several social behaviors. However, several variants are present at most sites and only lacking at a few sites. Consequently, if we only compared a subset of the number of populations, we would capture far fewer cultural variants (Figure 1). More importantly, the trajectory of the curve in Figure 1 suggests that, even by including all seven sites in the comparison, we are far from capturing the true chimpanzee cultural repertoire. Adding more populations into the comparison would probably lead to a further increase in the number of cultural variants. Accordingly, the repertoires obtained by the MoE are inherently incomplete.

The MoE is, without doubt, a great tool to detect likely cultural variation between populations. However, using geographic variation as the defining feature of culture makes it impossible to connect individual-level capacities to the trait. Furthermore, the MoE is a very poor technique to estimate either the size or the contents of cultural repertoires. If we applied this technique to humans, only a small and highly biased subset of what we naturally claim as part of our culture would remain, leaving out, for instance, most subsistence-related behaviors. Consequently, the lists of cultural variants described for non-human animal species in previous studies potentially vastly underestimate the actual sizes of their cultural repertoires.

## Describing Animal Culture without Relying on Geographic Variation

To avoid the biases of the MoE, and capture culture irrespective of geographic variation, we here propose a novel way to estimate animal culture by directly capturing the process of cultural transmission, namely social learning. We propose to first identify and validate indicators of forms of social learning as actual means of social learning in a given species and then count the behaviors for which we can document that individuals deploy these indicators during skill acquisition. We call this procedure the method of counting socially learned skills (SLS).

### Counting socially learned skills in orangutans

We test the SLS on wild orangutans, in which social interactions (including incidents of social learning) are rather easy to quantify because of the species’ low level of sociability (van Schaik, 1999). Recently, we studied peering behavior (i.e. attentive and sustained close-range watching of the activities of a conspecific) in immature orangutans (*Pongo* sp.; Schuppli *et al.*, 2016a). We found strong evidence that peering by immatures is an expression of socially induced learning, since it was followed by selective practice, decreased with growing competence of the peerer and was more and more directed at less familiar role models with increasing age of the immature peerers. Furthermore, peering rates increased with increasing rarity and complexity of the observed behavior.

**Figure 2.**
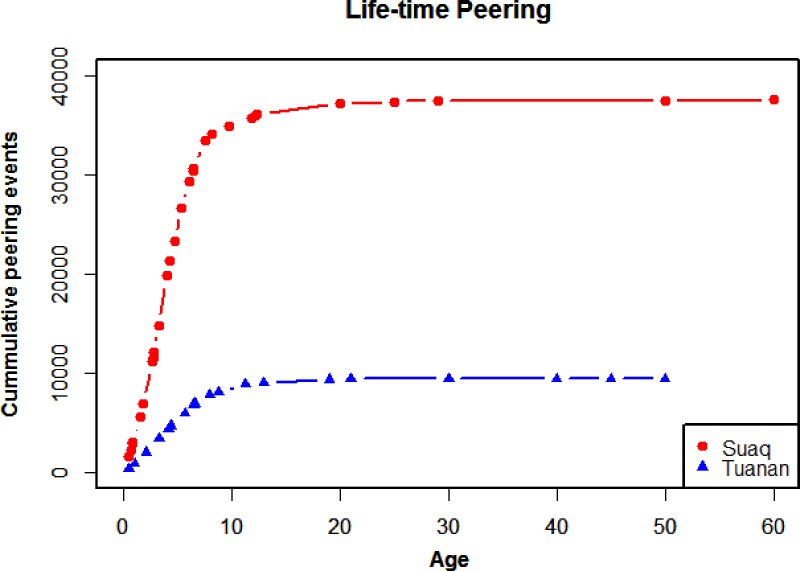
Lifetime orangutan peering. Extrapolated cumulative peering events over different ages for individuals at Suaq and Tuanan. See supplementary Table S1 for details.

Having validated peering as an index of social learning, we will first estimate the prevalence of social learning in orangutans by looking at how often individuals peer over their life time. By extrapolating cumulative peering counts based on known peering rates at different ages, we found that, over the course of their lives, Sumatran and Bornean orangutans (*Pongo abelii* and *Pongo pygmaeus wurmbi*) at the Suaq and Tuanan research sites peer around 38,000 and 9000 times (Figure 2). Individual's rates of peering are reduced to a minimum as adulthood is reached, at age 15.

**Figure 3.**
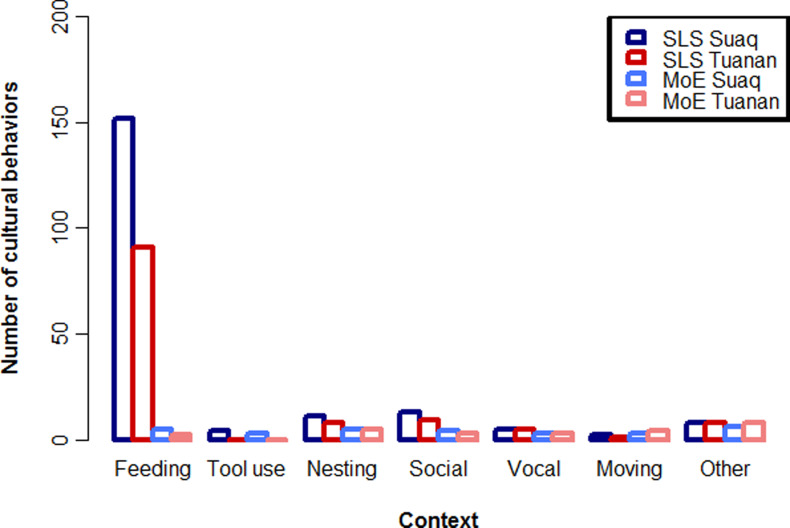
Comparing two methods to describe cultural repertoires. Number of cultural behaviors and knowledge elements caught by counting socially learned skills (SLS) vs relying on the MoE for the two orangutan populations at Suaq and Tuanan. See supplementary Tables S2 and S3 for the lists of peered at behaviors and behaviors caught by the MoE.

When we documented the peered-at behaviors our results showed that immatures peered for 195 and 122 different variants, including skills and knowledge elements (i.e. behavioral expressions of knowledge which shows no distinct skill, e.g. diet repertoires which are based on knowing that a certain fruit is edible), which we designated as probably SLS (Figure 3). This number is far greater than the 29 and 25 variants recognized by the MoE (van Schaik *et al.*, 2009). Thus, when looking closely at great ape skill acquisition, it seems that immatures learn virtually all of their skills socially.

When we compare the repertoires captured by the SLS and MoE (Figure 3) it becomes apparent that they also differ in the composition of the resulting culture catalogs. The SLS detected most socially learned skills in the foraging domain (food items and feeding techniques), followed by nesting behaviors, social behaviors, moving habits, tool use variants and other behaviors. The MoE produced a more equal distribution of SLS over these different categories. Moreover, the variants captured by the MoE were mainly conspicuous high-complexity behaviors, which are easy to notice for the human observer because they contain multiple steps of distinct, coordinated actions (in the case of Suaq, three tool use variants and five other high-complexity feeding techniques which contain multiple steps of processing, three distinct and loud vocalizations and four distinct social behaviors). The SLS list, in contrast, was mainly composed of basic feeding, nesting, and social skills. Most of the peered-at behaviors showed clear ecological correlates (e.g. food items that are only present at one site).

## Comparing the Two Methods to Capture Animal Culture

**Figure 4.**
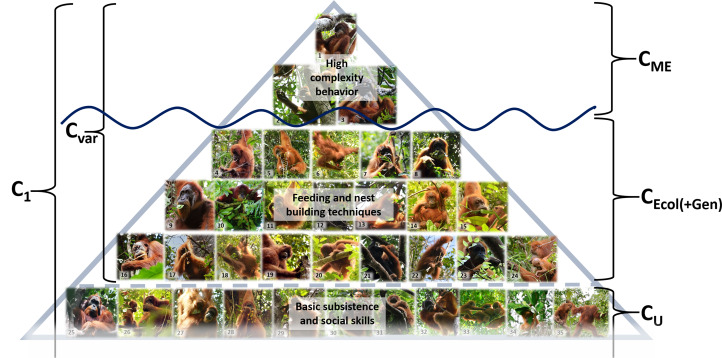
Different operationalizations of culture. The cultural behaviors captured by the MoE (*C*_ME_) are socially learned behaviors with a patchy geographic distribution but without ecological correlates (mostly conspicuous and/or high complexity behaviors such as tool use). The cultural behaviors with ecological correlates (*C*_Ecol_) are socially learned behaviors that vary between populations because they are influenced by a population's local ecology (e.g. feeding skills). The sum of *C*_ME_ and *C*_Ecol_ are all socially learned behaviors that vary across populations (*C*_Var_). Cultural universals (*C*_U_) are socially learned behaviors and knowledge that we find consistently across populations (e.g. basic subsistence and social skills). The sum of all socially learned behaviors represents an individual's cultural knowledge (*C*_1_ = *C*_Var_ + *C*_U_). See supplementary Table S3 descriptions of the behaviors depicted on the pictures.

According to the minimal definition of culture (Boyd and Richerson, 1985), an individual's cultural repertoire is the sum of all socially learned skills and knowledge (*C*_1_=∑SLS; Figure 3). According to the population-level concept of culture (culture as all behaviors that show interpopulation geographic variation), it is the sum of all SLS minus the sum of all cultural behaviors shared between the compared populations (*C*_Var_ = ∑*C*_1_ − ∑*C*_U_, Figure 4). The difference between these two measures of cultural repertoire sizes is, among other factors, dependent on the innovation rate in relation to transmission efficiency.

The cultural repertoires recorded by the MoE should be a direct subset of the sum of all socially learned behaviors that show geographic variation because they disregard any variants with ecological or genetic correlates. We will disregard genetic predispositions, because linking single behaviors to specific genes remains impossible, most likely because such direct links do not exist. The strength of ecological influences on culture is a positive function of the difference we get from subtracting the cultural repertoires captured by the MoE (*C*_ME_) from the sum of all socially learned behaviors that show geographic variation (*C*_Var_), i.e. *C*_Var_ – *C*_ME_ = ∑*C*_Ecol_ (Figure 4). A large difference implies that a lot of cultural variation is ecologically induced. Among orangutans, for instance, we find that many of their socially learned behaviors do indeed show ecological correlates such as food-processing skills or diet knowledge, which are highly dependent on the local availability of plant species.

So far, the effects of ecology on cultural variation have not yet been properly investigated. However, what has been shown is that ecological conditions have a strong effect on the two cornerstones of culture, namely innovation as the source of all culture and social learning as the underlying transmission mechanism of culture. First, innovation rates are heavily influenced by local ecology in terms of ecological opportunities (Koops *et al.*, 2014; Reader *et al.*, 2016; Spagnoletti *et al.*, 2012; Torralvo *et al.*, 2017). Second, social learning is influenced by ecological conditions such as terrestriality (Heldstab *et al.*, 2016; Meulman *et al.*, 2012; Sanz and Morgan, 2013; Spagnoletti *et al.*, 2009; Visalberghi *et al.*, 2005) or ecologically induced levels of sociability (Liker and Bókony, 2009; Roberts, 1996; Schuppli *et al.*, 2017; van Schaik *et al.*, 2003b). Furthermore, especially in long-lived species, increased learning efficiency through social learning is an important strategy to flexibly adapt to changing environments with local adaptations (Byrne *et al.*, 2004; Laland and Janik, 2006). As such, cultural capacity is highly adaptive.

To assess repertoire sizes, the SLS may be a great improvement over the MoE, but it may still miss cultural variants. Indeed, only around half of the orangutan cultural variants detected by the MoE were also captured by the SLS (18 of 29 for Suaq and 12 of 25 for Tuanan). The cultural variants for which we have no recorded peering events include behaviors whose transmission does not rely on peering (vocalizations and social interactions) or which are rarely performed. At both sites the lists of peered-at behaviors are still growing, despite massive follow efforts (around 19,000 hours for Suaq and 82,000 hours for Tuanan). It takes thousands of observation hours to capture an individual's complete behavioral repertoire (Schuppli *et al.*, 2016b). Also, even immatures who are still learning do not peer on every occasion of a behavior to be learned and we certainly miss a share of the peering events. In short, the current numbers still underestimate the real extent of the orangutan cultural repertoire.

## Discussion

### The base of the great ape culture iceberg

The orangutan example suggests that by relying on the MoE to assess cultural repertoires we have so far only discovered the tip of the great ape culture iceberg (i.e. *C*_1_ >> C_ME_; Figure 4). The MoE produces a biased sample of highly complex and conspicuous behaviors and dismisses a vast array of socially learned behaviors that covary with ecological factors. By counting socially learned skills, however, we are beginning to get to know the base of this iceberg. Cultural repertoires are mainly composed of basic, low-complexity subsistence skills, most of which show clear ecological correlates (e.g. knowledge of diet composition and processing techniques). Thus, a lot of (but not all) cultural variation may indeed be ecologically induced (*C*_Ecol_ is a major part of *C*_1_ and *C*_Var_).

At the same time, a systematic reliance on social learning under similar ecological conditions may very well lead to many universal cultural behavior patterns across populations. The most striking example in the orangutans for this is nest building: even though it is an orangutan universal, it takes young orangutans years of close observation and subsequent practice before they can build nests good enough to spend the night in (Schuppli *et al.*, 2016a), and socially deprived young apes will never be able to do so (Bernstein, 1962; Videan, 2006). The basic construction of nests (a rim made of intertwined long branches) is highly comparable across different orangutan populations, presumably because it is the most latent solution to the problem (Tennie *et al.*, 2009; high *C*_U_ but low *C*_Ecol_).

### How much culture is there in other animals?

The points discussed above are unlikely to be true only for orangutans or great apes in general but most certainly apply to all species that rely on social learning. Although numerous species, including insects, fish, birds and mammals, are now known to be capable of social learning (reviewed by Galef and Laland, 2005; Rapaport and Brown, 2008; Reader and Biro, 2010; Whiten, 2017), for most, social learning has so far only been shown in captivity, which does not elucidate to what extent species indeed use this ability in the wild (Reader and Biro, 2010; Whiten and van de Waal, 2018). Even though behavioral scientists now increasingly acknowledge the role of social learning (van Schaik and Burkart, 2011; Tomasello, 1999; van Schaik *et al.*, 2017), it is still widely treated as the rare and complex exception under the skill acquisition modes.

However, social learning can be quite simple given that many forms of social learning (e.g. enhancement or facilitation) do not require higher forms of cognition but nonetheless produce faithful behavioral copies owing to shared affordances. Furthermore, from the perspective of naïve immatures, a strong reliance on social learning is highly adaptive because social learning is less dangerous and more efficient than independent learning: it reduces the risk of getting injured or poisoned, increases learning speed by allowing the learning individual to benefit from what others have figured out before and increases the signal strength of relevant information (van Schaik and Burkart, 2011). Social learning thus allows for the fast acquisition of skills and the acquisition of more complex skills, and naïve individuals will benefit from choosing this option whenever they can. As such, we expect social learning to be most prominent in species with contact between generations, high social tolerance toward immatures, and an extended period of immaturity.

Over the last two decades it has become increasingly clear that social learning is indeed an important means of natural skill acquisition for many mammal and bird species, as evidenced in inherited dietary specializations, selective observations of skilled individuals, master apprentice interactions, effects of the presence of role models on foraging success or links between social networks and skill repertoires (Coelho *et al.*, 2015; Estes *et al.*, 2003; Griesser and Suzuki, 2016; Guinet and Bouvier, 1995; Hobaiter *et al.*, 2014; Kitowski, 2009; Krutzen *et al.*, 2005; Lonsdorf, 2006; Mann *et al.*, 2007; Matsuzawa *et al.*, 2001; Ottoni *et al.*, 2005; Rapaport and Brown, 2008; Schuppli *et al.*, 2016a). Direct observations of the spread of recently made innovations through social groups are bound to be rare but have been made in natural populations (Allen *et al.*, 2013; Hobaiter *et al.*, 2014; Kendal *et al.*, 2010). Interspecific cross-fostering experiments, be they designed or accidental, although both quite rare, have impressively demonstrated the pervasiveness of social learning of life's skills (Rowley and Chapman, 1986; Sheppard *et al.*, 2018; Slagsvold and Wiebe, 2007; Warner, 1988).

Culture is therefore likely to be pervasive in all species that pass on knowledge and skills socially. However, most of these species’ skills will show little or no geographic variation, except for the most complex skills, which are the least likely to be invented and retained. In several species, the acquisition of basic foraging skills was shown to be socially mediated: in aye-ayes (*Daubentonia madagascariensis*), for example, immatures learn tap-foraging – for which they even have morphological specializations – far less readily in the absence of adult role models (Krakauer, 2005).

Since social learning can be very simple, culture does not require a large brain and it is therefore unlikely to be a hallmark of cognitive complexity (Byrne *et al.*, 2004; Laland and Hoppitt, 2003), although the efficiency of cultural transmission may also favor the evolution of greater investment in brains (van Schaik and Burkart, 2011).

### Remaining challenges in the animal culture debate

Detecting animal culture irrespective of geographic variation is challenging and may not always be possible. Aside from peering, social learning can also happen via observation at longer distances, socially induced encounters with environmental features and acoustic transmission. Thus, in order to be able to draw conclusions about and compare cultural repertoires across species, it is crucial to find appropriate ways to detect social learning according to the species’ main transmission mode as well as to take different transmission modes into account. The SLS will thus most likely only rarely produce integral cultural repertoires. In most cases, however, it will be able to lift a significant part of the so far hidden base of the culture iceberg above the surface.

### Implications for human culture

Most elements which we nowadays naturally call the product of human culture can be found across the globe and are thus human universals. In this time of increasing connectedness and global exchange even the most complex human innovations often quickly reach the status of universals and would not be recognized as socially learned innovations by their geographic distribution. Yet everyone would agree that these innovations are an important part of our cultural repertoire.

What differentiates animal from human culture is the lack of normativity, the virtual absence of cumulative culture and the enormous diversity of human cultural elements (Laland and Galef, 2009; Whiten, 2017; Whiten and van Schaik, 2007). These three features seem to remain a hallmark of human culture and seem to be linked to the evolution of our species’ skill-intensive, technology-dependent foraging niche (van Schaik *et al.*, 2019; Laland, 2017). However, the unique human cultural constellation was built on a surprisingly broad and evolutionarily deep foundation.

## Conclusions

In sum, the true scope of animal culture will always be hard to estimate, but the most likely scenario is that what we have discovered so far using the MoE is only the tip of the animal cultural iceberg. Given that for most species social learning is the default mode of learning rather than the exception, repertoires of cultural behaviors are very likely to exceed current estimates. A wide spread of culture is in line with the notion that cultural capacity is an important tool that allows species to flexibly adapt to changing environments. If we intend to engage in a fair comparison with humans, we have to list a species’ complete repertoire of socially learned behaviors. Given that the right technique to capture social learning can be developed (dependent on the modality of social learning of each species), we will be able to compare the size and content of cultural repertoires across species. In the case of the great apes, culture seems to pervade virtually all aspects of their life, making them fundamentally as cultural as humans, just not cumulative, complex or normative.

## Data Availability

All data used for the analyses of this article can be found in the Supplementary Material (Tables S1–S3) and the published literature (indicated in the manuscript).

## Supplementary material

For supplementary material accompanying this paper visit http://dx.doi.org/10.1017/ehs.2019.1. click here to view supplementary material

## References

[ref1] Allen J, Weinrich M, Hoppitt W and Rendell L (2013). Network-based diffusion analysis reveals cultural transmission of lobtail feeding in humpback whales. Science 340(6131), 485–488. 23620054 10.1126/science.1231976

[ref2] Bernstein IS (1962). Response to nesting materials of wildborn and captive-born chimpanzees. Animal Behaviour 10(1–2), 1–6.

[ref3] Boyd R and Richerson PJ (1985). Culture and the Evolutionary Process. Chicago: University of Chicago Press.

[ref4] Braidwood RJ (1975). Prehistoric Men. Glenview: Scott Foresman.

[ref5] Byrne RW, Barnard PJ, Davidson I, Janik VM, McGrew WC, Miklosi A and Wiessner P (2004). Understanding culture across species. Trends in Cognitive Sciences 8(8), 341–346. 15335460 10.1016/j.tics.2004.06.002

[ref6] Catchpole CK and Slater PJ (2003). Bird Song: Biological Themes and Variations. Cambridge: Cambridge University Press.

[ref7] Coelho CG, Falotico T, Izar P, Mannu M, Resende BD, Siqueira JO and Ottoni EB (2015). Social learning strategies for nut-cracking by tufted capuchin monkeys (*Sapajus* spp.). Animal Cognition 18(4), 911–919, https://doi:10.1007/s10071-015-0861-5 25800169 10.1007/s10071-015-0861-5

[ref8] Estes JA, Riedman ML, Staedler MM, Tinker MT and Lyon BE (2003). Individual variation in prey selection by sea otters: patterns, causes and implications. Journal of Animal Ecology 72(1), 144–155, https://doi:10.1046/j.1365-2656.2003.00690.x

[ref9] Fragaszy D and Perry S (2003). Towards a biology of traditions. In Fragaszy D and Perry S (eds), The Biology of Traditions: Models and Evidence. Cambridge: Cambridge University Press, pp. 1–32.

[ref10] Galef BG Jr (1976). Social transmission of acquired behavior: a discussion of tradition and social learning in vertebrates. In Rosenblatt JS, Hinde RA, Shaw E and Beer C (eds), Advances in the Study of Behavior. New York: Academic Press, Vol. 6, pp. 77–100.

[ref11] Galef BG Jr (1992). The question of animal culture. Human Nature 3, 157–178. 24222403 10.1007/BF02692251

[ref12] Galef BG and Laland KN (2005). Social learning in animals: empirical studies and theoretical models. Bioscience 55(6), 489–499, https://doi:10.1641/0006-3568(2005)055[0489:sliaes]2.0.co;2

[ref13] Griesser M and Suzuki TN (2016). Kinship modulates the attention of naive individuals to the mobbing behaviour of role models. Animal Behaviour 112, 83–91, https://doi:10.1016/j.anbehav.2015.11.020

[ref14] Guinet C and Bouvier J (1995). Development of intentional stranding hunting techniques in killer whale (*Orcinus-Orca*) calves at Crozet archipelago. Canadian Journal of Zoology – Revue Canadienne De Zoologie 73(1), 27–33, https://doi:10.1139/z95-004

[ref15] Heldstab SA, Kosonen ZK, Koski SE, Burkart JM, van Schaik CP and Isler K (2016). Manipulation complexity in primates coevolved with brain size and terrestriality. Scientific Reports 6, 24528, https://doi:10.1038/srep24528 27075921 10.1038/srep24528 PMC4830942

[ref16] Heyes C (2012). What's social about social learning? Journal of Comparative Psychology 126(2), 193–202, https://doi:10.1037/a0025180 21895355 10.1037/a0025180

[ref17] Hill KR (2009). Animal ‘culture’. In Laland KN and Galef BG (eds), The Question of Animal Culture. Cambridge, MA: Harvard University Press, pp. 269–287.

[ref18] Hobaiter C, Poisot T, Zuberbuehler K, Hoppitt W and Gruber T (2014). Social network analysis shows direct evidence for social transmission of tool use in wild chimpanzees. Plos Biology 12(9), https://doi:10.1371/journal.pbio.1001960 10.1371/journal.pbio.1001960 PMC4181963 25268798

[ref19] Hohmann G and Fruth B (2003). Culture in bonobos? Between-species and within-species variation in behavior. Current Anthropology 44(4), 563–571.

[ref20] Kendal RL, Custance DM, Kendal JR, Vale G, Stoinski TS, Rakotomalala NL and Rasamimanana H (2010). Evidence for social learning in wild lemurs (*Lemur catta*). Learning & Behavior 38(3), 220–234. 20628161 10.3758/LB.38.3.220

[ref21] Kitowski I (2009). Social learning of hunting skills in juvenile marsh harriers *Circus aeruginosus*. Journal of Ethology 27(3), 327–332, https://doi:10.1007/s10164-008-0123-y

[ref22] Koops K, Visalberghi E and van Schaik CP (2014). The ecology of primate material culture. Biology Letters 10, 20140508. 25392310 10.1098/rsbl.2014.0508 PMC4261853

[ref23] Krakauer EB (2005). Development of Aye-Aye (Daubentonia madagascariensis) Foraging Skills: Independent Exploration and Social Learning. Durham NC: Duke University.

[ref24] Krutzen M, Mann J, Heithaus MR, Connor RC, Bejder L and Sherwin WB (2005). Cultural transmission of tool use in bottlenose dolphins. Proceedings of the National Aacademy of Sciences USA 102(25), 8939–8943, https://doi:10.1073/pnas.0500232102 10.1073/pnas.0500232102 PMC1157020 15947077

[ref25] Krützen M, Willems EP and van Schaik CP (2011). Culture and geographic variation in orangutan behavior. Current Biology 21, 1808–1812. 22018539 10.1016/j.cub.2011.09.017

[ref26] Laland KN (2017). Darwin's Unfinished Symphony: How Culture Made the Human Mind. Princeton, NJ: Princeton University Press.

[ref27] Laland KN and Galef BG (2009). The Question of Animal Culture. Cambridge, MA: Harvard University Press.

[ref28] Laland KN and Hoppitt W (2003). Do animals have culture? Evolutionary Anthropology 12, 150–159.

[ref29] Laland KN and Janik VM (2006). The animal cultures debate. Trends in Ecology and Evolution 21, 542–547. 16806574 10.1016/j.tree.2006.06.005

[ref30] Langergraber KE and Vigilant L (2011). Genetic differences cannot be excluded from generating behavioural differences among chimpanzee groups. Proceedings of the Royal Society B: Biological Sciences 278(1715), 2094–2095.

[ref31] Langergraber KE, Boesch C, Inoue E, Inoue-Murayama M, Mitani JC, Nishida T, Wrangham RW (2010). Genetic and ‘cultural’ similarity in wild chimpanzees. Proceedings of the Royal Society B: Biological Sciences 278(1704), 408–416. 10.1098/rspb.2010.1112 PMC3013405 20719777

[ref32] Levinson SC (2006). Introduction: The Evolution of Culture in a Microcosm, Levinson SC and Jaisson ((eds). Cambridge, MA: MIT Press.

[ref33] Liker A and Bókony V (2009). Larger groups are more successful in innovative problem solving in house sparrows. Proceedings of the National Academy of Sciences 106(19), 7893–7898, https://doi:10.1073/pnas.0900042106 10.1073/pnas.0900042106 PMC2683070 19416834

[ref34] Lonsdorf E (2006). What is the role of mothers in the acquisition of termite-fishing behaviors in wild chimpanzees (*Pan troglodytes schweinfurthii*)? Animal Cognition 9(1), 36–46. 16195914 10.1007/s10071-005-0002-7

[ref35] Lycett SJ, Collard M and McGrew WC (2009). Cladistic analyses of behavioural variation in wild *Pan troglodytes*: exploring the chimpanzee culture hypothesis. Journal of Human Evolution 57, 337–349. 19762070 10.1016/j.jhevol.2009.05.015

[ref36] Lycett SJ, Collard M and McGrew WC (2011). Correlations between genetic and behavioural dissimilarities in wild chimpanzees (*Pan troglodytes*) do not undermine the case for culture. Proceedings of the Royal Society B: Biological Sciences 278(1715), 2091–2093. 10.1098/rspb.2010.2599 PMC3107635 21490014

[ref37] Mann J, Sargeant BL and Minor M (2007). Calf inspections of fish catches in bottlenose dolphins (*Tursiop*s sp.): opportunities for oblique social learning? Marine Mammal Science 23(1), 197–202, https://doi:10.1111/j.1748-7692.2006.00087.x

[ref38] Matsuzawa T, Biro D, Humle T, Inoue-Nakamura N, Tonooka R and Yamakoshi G (2001). Emergence of culture in wild chimpanzees: education by master-apprenticeship. In Matsuzawa T (ed.), Primate Origins of Human Cognition and Behavior. Tokyo: Springer, pp. 557–574.

[ref39] McGrew WC (2004). The Cultured Chimpanzee: Reflections on Cultural Primatology. Cambridge: Cambridge University Press.

[ref40] McGrew WC and Tutin CEG (1978). Evidence for a social custom in wild chimpanzees? Man 13, 234–251.

[ref41] Meulman EJ, Sanz CM, Visalberghi E and van Schaik CP (2012). The role of terrestriality in promoting primate technology. Evolutionary Anthropology 21(2), 58–68. 22499440 10.1002/evan.21304

[ref42] Nishida T (1987). Local traditions and cultural transmission. In Smuts BB, Cheney DL, Seyfarth RM, Wrangham RW and Struhsaker TT (eds), Primate Societies. Chicago, IL: University of Chicago Press, pp. 462–474.

[ref43] Ottoni E and Izar P (2008). Capuchin monkey tool use: overview and implications. Evolutionary Anthropology 17, 171–178.

[ref44] Ottoni EB, de Resende BD and Izar P (2005). Watching the best nutcrackers: what capuchin monkeys (*Cebus apella*) know about others' tool-using skills. Animal Cognition 8(4), 215–219, https://doi:10.1007/s10071-004-0245-8 15719240 10.1007/s10071-004-0245-8

[ref45] Perry S (2009a). Are nonhuman primates likely to exhibit cultural capacities like those of humans? In Laland KN and Galef BG (eds), The Question of Animal Culture. Cambrdige, MA: Harvard University Press, pp. 247–268.

[ref46] Perry S (2009b). Socia influence and the development of food processing techniques in wild white-faced cauchin monkeys (*Cebus capucinus*) at Lomas Barbudal, Costa Rica. American Journal of Primatology 71, 99–99.

[ref47] Perry S, Baker M, Fedigan L, Gros-Louis J, Jack K, Mackinnon KC, Rose LM (2003). Social conventions in wild white-faced capuchin monkeys: evidence for traditions in a neotropical primate. Current Anthropology 44, 214–268.

[ref48] Rapaport LG and Brown GR (2008). Social influences on foraging behavior in young nonhuman primates: learning what, where, and how to eat. Evolutionary Anthropology 17(4), 189–201, https://doi:10.1002/evan.20180

[ref49] Reader SM and Biro D (2010). Experimental identification of social learning in wild animals. Learning & Behavior 38(3), 265–283, https://doi:10.3758/lb.38.3.265 20628165 10.3758/LB.38.3.265

[ref50] Reader SM, Morand-Ferron J and Flynn E (2016). Animal and human innovation: novel problems and novel solutions. Philosophical Transactions of the Royal Society B: Biological Sciences 371, 20150182. 10.1098/rstb.2015.0182 PMC4780525 26926273

[ref51] Riebel K, Lachlan RF and Slater PJ (2015). Learning and cultural transmission in chaffinch song Advances in the Study of Behavior. Oxford: Elsevier, Vol. 47, pp. 181–227.

[ref52] Robbins MM, Ando C, Fawcett KA, Grueter CC, Hedwig D, Iwata Y, Stoinski TS (2016). Behavioral variation in gorillas: evidence of potential cultural traits. PloS One 11(9), e0160483. 27603668 10.1371/journal.pone.0160483 PMC5014408

[ref53] Roberts G (1996). Why individual vigilance declines as group size increases. Animal Behaviour 51, 1077–1086, https://doi:10.1006/anbe.1996.0109

[ref54] Rowley I and Chapman G (1986). Cross-fostering, imprinting and learning in two sympatric species of cockatoo. Behaviour 96, 1–16.

[ref55] Santorelli CJ, Schaffner CM, Campbell CJ, Notman H, Pavelka MS, Weghorst JA and Aureli F (2011). Traditions in spider moonkeys are biased towards the social domain. PloS One 6(2), e16863. 21373196 10.1371/journal.pone.0016863 PMC3044143

[ref56] Sanz CM and Morgan DB (2013). Ecological and social correlates of chimpanzee tool use. Philosophical Transactions of the Royal Society B: Biological Sciences 368(1630), 20120416. 10.1098/rstb.2012.0416 PMC4027411 24101626

[ref57] Schuppli C and van Schaik C (2019). Social learning among wild orangutans: is it affective? In Clément F and Dukes D (eds), Foundations of Affective Social Learning: Conceptualising the Transmission of Social Value. Cambridge: Cambridge University Press, in press.

[ref58] Schuppli C, Meulman E, Forss SIF, Aprilinayati F, Van Noordwijk MA and Van Schaik CP (2016a). Observational social learning and socially induced practice of routine skills in wild immature orang-utans. Animal Behaviour 119, 87–98, https://doi:10.1016/j.anbehav.2016.06.014

[ref59] Schuppli C, Forss SI, Meulman EJ, Zweifel N, Lee KC, Rukmana E, van Schaik CP (2016b). Development of foraging skills in two orangutan populations: needing to learn or needing to grow? Frontiers in Zoology 13(1), 43. 27708679 10.1186/s12983-016-0178-5 PMC5041519

[ref60] Schuppli C, Forss S, Meulman E, Atmoko SU, Noordwijk M and Schaik C (2017). The effects of sociability on exploratory tendency and innovation repertoires in wild Sumatran and Bornean orangutans. Scientific Reports 7(1), 15464. 29133851 10.1038/s41598-017-15640-x PMC5684228

[ref61] Sheppard CE, Marshall HH, Inger R, Thompson FJ, Vitikainen EI, Barker S, Nichols HJ, Wells DA, McDonald RA and Cant MA (2018). Decoupling of genetic and cultural inheritance in a wild mammal. Current Biology 28(11), 1846–1850. 29804813 10.1016/j.cub.2018.05.001

[ref62] Slagsvold T and Wiebe KL (2007). Learning the ecological niche. Proceedings of the Royal Society B 274, 19–23. 17015332 10.1098/rspb.2006.3663 PMC1679873

[ref63] Spagnoletti N, Izar P and Visalberghi E (2009). Tool use and terrestriality in wild bearded capuchin monkey (*Cebus libidinosus*). Folia Primatologica 80(2), 142–142.

[ref64] Spagnoletti N, Visalberghi E, Verderane MP, Ottoni E, Izar P and Fragaszy D (2012). Stone tool use in wild bearded capuchin monkeys, *Cebus libidinosus*. Is it a strategy to overcome food scarcity? Animal Behaviour 83(5), 1285–1294.

[ref65] Tennie C, Call J and Tomasello M (2009). Ratcheting up the ratchet: on the evolution of cumulative culture. Philosophical Transactions of the Royal Society B – Biological Sciences 364(1528), 2405–2415, https://doi:10.1098/rstb.2009.0052 10.1098/rstb.2009.0052 PMC2865079 19620111

[ref66] Tomasello M (1999). The Cultural Origins of Human Cognition. Cambridge MA: Harvard University Press.

[ref67] Tomasello M, Krufer A and Ratner H (1993). Cultural learning. Behavioral and Brain Science 16, 495–511.

[ref68] Torralvo K, Rabelo RM, Andrade A and Botero-Arias R (2017). Tool use by Amazonian capuchin monkeys during predation on caiman nests in a high-productivity forest. Primates 58(2), 279–283. 28281099 10.1007/s10329-017-0603-1

[ref69] Tylor EB (1871). Primitive Culture: Researches into the Development of Mythology, Philosophy, Religion, Art, and Custom. London: J. Murray, Vol. 2.

[ref70] van Schaik CP (1999). The socioecology of fission–fusion sociality in orangutans. Primates 40, 73–90. 10.1007/BF02557703 23179533

[ref71] van Schaik CP (2010). Social learning and culture in animals. In Kappeler P (ed.), Animal Behaviour: Evolution and Mechanisms. Heidelberg, Berlin: Springer, pp. 623–653.

[ref72] van Schaik CP (2016). The Primate Origins of Human Nature. Chichester: John Wiley & Sons, Vol. 2.

[ref73] van Schaik CP and Burkart JM (2011). Social learning and evolution: the cultural intelligence hypothesis. Royal Society Philosophical Transactions Biological Sciences 366(1567), 1008–1016. 10.1098/rstb.2010.0304 PMC3049085 21357223

[ref74] van Schaik CP, Ancrenaz M, Borgen G, Galdikas B, Knott CD, Singleton I, Suzuki A, Utami SS and Merrill M (2003a). Orangutan cultures and the evolution of material culture. Science 299(5603), 102–105, https://doi:10.1126/science.1078004 12511649 10.1126/science.1078004

[ref75] van Schaik CP, Fox EA and Fechtman LT (2003b). Individual variation in the rate of use of tree-hole tools among wild orang-utans: implications for hominin evolution. Journal of human evolution 44(1), 11–23, https://doi:10.1016/s0047-2484(02)00164-1 12604301 10.1016/s0047-2484(02)00164-1

[ref76] van Schaik CP, Ancrenaz M, Reniastoeti D, Knott CD, Morrogh-Bernard H, Nuzuar OK, Atmoko S and Noordwijk MA (2009). Orangutan cultures revisited. In Wich SA, Mitra Setia T, Utami Atmoko SA and Van Schaik CP (eds.), Orangutans Compared: Geographic Variation in Behavioral Ecology and Conservation. Oxford: Oxford University Press, pp. 299–309.

[ref77] van Schaik CP, Graber S, Schuppli C and Burkart J (2017). The ecology of social learning in animals and its link with intelligence. The Spanish Journal of Psychology 19, 1–12. 10.1017/sjp.2016.100 28065213

[ref77a] van Schaik CP, Pradhan GR and Tennie C (2019). Teaching and curiosity: sequential drivers of cumulative cultural evolution in the hominin lineage. Behavioral ecology and sociobiology 73, 2.

[ref78] Videan EN (2006). Bed-building in captive chimpanzees (*Pan troglodytes*): the importance of early rearing. American Journal of Primatology 68, 745–751. 16786520 10.1002/ajp.20265

[ref79] Visalberghi E, Fragaszy DM, Izar P and Ottoni EB (2005). Terrestriality and tool use. Science 308(5724), 951–952; author reply 951–952. 10.1126/science.308.5724.951c 15890860

[ref80] Warner RR (1988). Traditionality of mating-site preferences in a coral reef fish. Nature 335, 719, https://doi:10.1038/335719a0

[ref81] Whitehead H and Rendell L (2014). The Cultural Lives of Whales and Dolphins. Chicago, IL: University of Chicago Press.

[ref82] Whiten A (2012). Social learning, traditions, and culture. In Mitani JC, Call J, Kappeler PM, Palombit RA and Silk JB (eds), The Evolution of Primate Societies, pp. 682–700.

[ref83] Whiten A (2017). Culture extends the scope of evolutionary biology in the great apes. *Proceedings of the National Academy of Sciences*, 201620733.

[ref84] Whiten A and Mesoudi A (2008). Establishing an experimental science of culture: animal social diffusion experiments. Philosophical Transactions of the Royal Society B – Biological Sciences 363(1509), 3477–3488, https://doi:10.1098/rstb.2008.0134 10.1098/rstb.2008.0134 PMC2607342 18799418

[ref85] Whiten A and van de Waal E (2018). The pervasive role of social learning in primate lifetime development. Behavioral Ecology and Sociobiology 72(5), 80. 29755181 10.1007/s00265-018-2489-3 PMC5934467

[ref86] Whiten A and van Schaik CP (2007). The evolution of animal ‘cultures’ and social intelligence. Philosophical Transactions of the Royal Society B – Biological Sciences 362(1480), 603–620, https://doi:10.1098/rstb.2006.1998 10.1098/rstb.2006.1998 PMC2346520 17255007

[ref87] Whiten A, Goodall J, McGrew WC, Nishida T, Reynolds V, Sugiyama Y, Tutin CEG, Wrangham RW and Boesch C (1999). Cultures in chimpanzees. Nature 399(6737), 682–685, https://doi:10.1038/21415 10385119 10.1038/21415

