# Supplementary material for: Animal cultures: how we've only seen the tip of the iceberg
Source: Evol Hum Sci. 2019 May 23;1:e2. doi: 10.1017/ehs.2019.1 (PMC10427297; doi:10.1017/ehs.2019.1)
Supplement: Supplementary file 1 [file S2513843X1900001Xsup001.docx]

**Supplementary material**

**Table S1. Orangutan life-time peering.** Cumulative peering events (Peering cumulative) were calculated via peering rates (average number of peering events per observation hour) obtained during all occurrence data collection over an extended observation time whereby each age-individual data point was based on data collected within maximally 5 months (see (Schuppli et al., 2016) for detailed methods). Daily peering rates (Peering/ day) were calculated with an average day length of 11.5 hours. Peering events per phase (Peering/ phase) were calculated using the average peering rate of that age phase times the phase length. Cumulative peering events were calculated by summing up the peering events of the phases. Site: Tua= Tuanan, Sua= Suaq; Class: Im= immature, Ad= adult, Sex: F= female, M= male.

| **Focal** | **Site** | **Class** | **Sex** | **Age** | **Observation time (h)** | **Peering rate** | **Peering/ day** | **Peering/ phase** | **Peering cumulative** |
| --- | --- | --- | --- | --- | --- | --- | --- | --- | --- |
| Lois | Sua | Im | M | 0.5 | 110.8 | 1.40 | 16.08 | 1556.67 | 1557 |
| Rendang | Sua | Im | M | 0.7 | 39.9 | 0.75 | 8.64 | 722.40 | 2279 |
| Simba | Sua | Im | M | 0.9 | 59.3 | 0.71 | 8.14 | 612.93 | 2892 |
| Frankie | Sua | Im | M | 0.9 | 110.5 | 0.88 | 10.09 | 66.60 | 2959 |
| Cinnamon | Sua | Im | F | 1.6 | 87.2 | 0.99 | 11.35 | 2544.82 | 5503 |
| Ronaldo | Sua | Im | M | 1.9 | 73.7 | 1.22 | 14.05 | 1391.53 | 6895 |
| Fredy | Sua | Im | M | 2.7 | 77.3 | 1.23 | 14.14 | 4222.32 | 11117 |
| Eden | Sua | Im | F | 2.8 | 64.1 | 0.73 | 8.43 | 412.32 | 11530 |
| Lois | Sua | Im | M | 2.9 | 83.4 | 1.83 | 21.09 | 539.17 | 12069 |
| Fredy | Sua | Im | M | 3.3 | 100 | 1.56 | 17.94 | 2708.74 | 14777 |
| Fredy | Sua | Im | M | 4 | 58.8 | 1.53 | 17.60 | 5063.39 | 19841 |
| Frankie | Sua | Im | M | 4.3 | 75 | 1.19 | 13.65 | 1484.05 | 21325 |
| Cinnamon | Sua | Im | F | 4.7 | 95.5 | 0.96 | 11.08 | 1987.28 | 23312 |
| Chindy | Sua | Im | F | 5.4 | 60.3 | 1.43 | 16.40 | 3362.40 | 26675 |
| Fredy | Sua | Im | M | 6.1 | 112.6 | 0.48 | 5.52 | 2601.62 | 29276 |
| Lois | Sua | Im | M | 6.4 | 72.4 | 1.01 | 11.60 | 1187.42 | 30464 |
| Lilly | Sua | Im | F | 6.5 | 99.1 | 0.84 | 9.64 | 193.87 | 30658 |
| Lilly | Sua | Im | F | 7.6 | 29.8 | 0.44 | 5.02 | 2837.28 | 33495 |
| Chindy | Sua | Im | F | 8.1 | 48.1 | 0.08 | 0.96 | 643.96 | 34139 |
| Lilly | Sua | Im | F | 9.8 | 77.9 | 0.14 | 1.62 | 763.67 | 34902 |
| Ellie | Sua | Im | F | 11.8 | 63.6 | 0.06 | 0.72 | 891.98 | 35794 |
| Shera | Sua | Im | F | 12.2 | 100.6 | 0.27 | 3.09 | 271.34 | 36066 |
| Lilly | Sua | Im | F | 12.3 | 59.6 | 0.07 | 0.77 | 77.51 | 36143 |
| Ellie | Sua | Ad | F | 20 | 64.1 | 0.00 | 0.00 | 1079.69 | 37223 |
| Sarabi | Sua | Ad | F | 25 | 59.3 | 0.02 | 0.19 | 176.99 | 37400 |
| Lisa | Sua | Ad | F | 29 | 266.7 | 0.00 | 0.00 | 141.59 | 37542 |
| Cissy | Sua | Ad | F | 50 | 230.8 | 0.00 | 0.00 | 0.00 | 37542 |
| Friska | Sua | Ad | F | 60 | 298.1 | 0.00 | 0.04 | 70.45 | 37612 |
| Kahiyu | Tua | Im | F | 0.5 | 114.8 | 0.27 | 3.11 | 286.09 | 286 |
| Joya | Tua | Im | M | 1.1 | 78.2 | 0.17 | 1.91 | 511.28 | 797 |
| Danum | Tua | Im | M | 2.1 | 122 | 0.35 | 4.05 | 1178.38 | 1976 |
| Joya | Tua | Im | M | 3.3 | 78 | 0.21 | 2.36 | 1404.60 | 3380 |
| Mawas | Tua | Im | F | 4.1 | 122.4 | 0.35 | 4.04 | 927.45 | 4308 |
| Petzy | Tua | Im | M | 4.4 | 49 | 0.20 | 2.35 | 338.86 | 4647 |
| Kino | Tua | Im | M | 5.7 | 109.2 | 0.27 | 3.05 | 1263.72 | 5910 |
| Mawas | Tua | Im | F | 6.5 | 78.5 | 0.28 | 3.22 | 876.94 | 6787 |
| Jip | Tua | Im | M | 6.6 | 140 | 0.25 | 2.88 | 129.78 | 6917 |
| Deri | Tua | Im | M | 8 | 53.7 | 0.04 | 0.43 | 848.39 | 7765 |
| Jip | Tua | Im | M | 8.8 | 74.6 | 0.12 | 1.39 | 266.69 | 8032 |
| Milo | Tua | Im | F | 11.3 | 97.7 | 0.03 | 0.35 | 794.45 | 8827 |
| Kondor | Tua | Ad | F | 13 | 114.8 | 0.02 | 0.20 | 172.48 | 8999 |
| Juni | Tua | Ad | F | 19 | 214.6 | 0.01 | 0.11 | 337.18 | 9336 |
| Desy | Tua | Ad | F | 21 | 122 | 0.00 | 0.00 | 39.17 | 9375 |
| Mindy | Tua | Ad | F | 30 | 200.9 | 0.00 | 0.00 | 0.00 | 9375 |
| Kerry | Tua | Ad | F | 40 | 109.2 | 0.00 | 0.00 | 0.00 | 9375 |
| Pinky | Tua | Ad | F | 45 | 49 | 0.00 | 0.00 | 0.00 | 9375 |
| Jinak | Tua | Ad | F | 50 | 156.2 | 0.00 | 0.00 | 0.00 | 9375 |

**Table S2.** **Orangutan cultural variants.** List of orangutan cultural variants retrieved from (van Schaik et al., 2009). Category: F= Feeding, M= moving, N= nesting, O= other behaviors, S= social interactions, T= tool use, V= vocalization. Present: 0= absent, 1= present (updated with our newest data for both sites), Peer: 0= no peering recorded, 1= at least one peering event recorded for the specific behavior.

| **Cultural variant** | **Category** | **Suaq** | | **Tuanan** | |
| --- | --- | --- | --- | --- | --- |
|  |  | **Present** | **Peer** | **Present** | **Peer** |
| c1 Snag riding: Ride on pushed-over snag as it falls, then grab on to vegetation before it crashes | M | 1 | 0 | 1 | 0 |
| c2 Kiss squeak with leaves: Using leaves on mouth to amplify sound, then drop leaf | V | 1 | 0 | 1 | 1 |
| c4 Leaf wipe: wiping face with fistful of squashed leaves, then drop (in kiss squeak context) | O | 0 |  | 0 |  |
| c6 Bunk nests: build a nest a short distance above the nest used for resting (during rain) | N | 1 | 1 | 1 | 0 |
| c7 Sun cover: building cover on nest during bright sunshine (rather than rain) | N | 1 | 0 | 1 | 0 |
| c8 Hide under nest: seek shelter under nest for rain | N | 1 | 1 | 1 | 0 |
| c9 Scratch with stick: using detached stick to scratch body parts | O | 1 | 0 | 1 | 0 |
| c10 Auto-erotic tool: using tool for sexual stimulation (female and male) | O | 0 |  | 0 |  |
| c11 Raspberry: spluttering sounds associated with nest building | V | 1 | 1 | 0 |  |
| c12 Symmetric scratch: exaggerated, long, slow, symmetric scratching movements with both arms at same time | O | 1 | 0 | 0 |  |
| c13 Twig biting: systematically passing ends of twigs used for lining of nest past the mouth (sometimes including actual bite) during last phase of nest building | N | 1 | 1 | 1 | 1 |
| c14 Leaf napkin: using handful of leaves to wipe latex of chin | O | 0 |  | 0 |  |
| c15 Branch as swatter: using detached leafy branches to ward off bees/wasps attacking subject (who is raiding their nest) | F | 1 | 1 | 0 |  |
| c16 Leaf gloves/cushions: using leaf gloves to handle spiny fruits or spiny branch, or as seat cushions in trees with spines | O | 0 |  | 0 |  |
| c17 Tree-hole tool-use: using tool to poke into tree holes to obtain social insects or their products | T | 1 | 1 | 0 |  |
| c18 Seed-extraction tool use: using tool to extract seeds from the protected fruits of Neesia sp. | T | 1 | 1 | 0 |  |
| c19 Branch scoop: drinking water from deep tree hole using leafy branch (water dripping from leaves) | F | 0 |  | 0 |  |
| c21 Bouquet feeding: using lips to pick ants from fistful of dry, or fresh, or rotting leaves (nests) | F | 1 | 1 | 1 | 1 |
| c23 Dead twig sucking: breaking hollow (dead) twigs to suck ants from inside | F | 1 | 1 | 0 |  |
| c24 Slow loris eating: capture and eat slow loris hiding in dense vegetation | F | 1 | 1 | 0 |  |
| c25 Nest smack: smacking sounds associated with nest building | V | 0 |  | 1 | 1 |
| c26 Carry leafy branch to different tree in which subject starts to build nest using carried leaves as lining, pillow or cover (Tuanan: Campnosperma) | N | 1 | 1 | 1 | 1 |
| c27 Branch cushion: covering (non-spiny) big branch(es) with few leaves or leafy branches, then sit or lie on it | O | 1 | 1 | 1 | 1 |
| c28 Throat scrape: deep throat sound made by female towards offspring | V | 1 | 0 | 1 | 1 |
| c29 Moss cleaning: cleaning hands with moss | O | 0 |  | 0 |  |
| c30 Chewing leaves into pulp then smearing foam over body (selected parts chosen) until fur all wet | O | 0 |  | 0 |  |
| c31 Male and female use the same nest to spend the entire night (during consortship) | S | 1 | 0 | 0 |  |
| c32 Tooth cleaning: chewing and spitting out leaves after eating (sticky?) fruit | O | 0 |  | 0 |  |
| c33 Copulation on female’s nest | S | 0 |  | 1 | 1 |
| c34 Using Asplenium fern to rest or sleep in | N | 0 |  | 0 |  |
| c35 Nest as social refuge: female on nest left alone by otherwise coercive male | S | 1 | 0 | 0 |  |
| c36(ex-i7) Biting through vine to swing Tarzan-style across gap | M | 1 | 1 | 1 | 0 |
| c37(ex-i13) Biting through vine to release tree for swaying to reach adjacent tree | M | 1 | 0 | 1 | 0 |
| c38(ex-i14) Coercive hand-holding: male firmly holds female’s hand/wrist for long period of time during consort | S | 1 | 1 | 1 | 1 |
| c39 (ex-i17) Water play (wild splashing) on ground | O | 0 |  | 1 | 1 |
| i1 GG rub: females rubbing their genitals together | S | 1 | 0 | 1 | 0 |
| i2 Use leaf to clean body surface | O | 0 |  | 0 |  |
| i3 Sneaky nest approach: building a series of nests, while approaching conspecific in fruit tree | S | 0 |  | 0 |  |
| i4 Leaf bundle (‘doll’): carried around and taken to nest | O | 0 |  | 1 | 0 |
| i5 Leaf scoop: drinking water from ground or stream using leaf as vessel (drinking directly from vessel) | F | 0 |  | 0 |  |
| i9 Branch dragging display on the ground | O | 0 |  | 0 |  |
| i11 Sponging: drinking water using crumpled leaves | F | 0 |  | 0 |  |
| i12 Hiding behind detached branch from predators or humans | O | 0 |  | 1 | 0 |
| i13 Stick as chisel (1) to open termite nest in log or ant nest on ground | T | 1 | 1 | 0 |  |
| i15 Branch hook: using detached branch to pull branch of adjacent tree to within reach | M | 0 |  | 1 | 0 |
| i18 Drink from bottom of pitcher plant after biting through bottom (not drink like cup!) | F | 1 | 1 | 1 | 1 |
| i19 Poultice use: chewing leaves and applying resulting poultice to wound | O | 0 |  | 0 |  |
| i20 Long-call vibrato: using fingers to make pulses at end of long call (fingers come over head onto lips) | V | 0 |  | 0 |  |
| i21 Use gloves to get into ants’ nest (and avoid being bitten) | F | 0 |  | 0 |  |
| i22 Tooth pick: use a small stick to clean teeth | O | 1 | 0 | 1 | 0 |
| i23 Nail cleaning: use small stick to clean under finger nail | O | 0 |  | 0 |  |
| i24 Washing face and arms with water from tree hole | O | 1 | 1 | 1 | 1 |
| i25 Stick as chisel (2) to open durian fruit | T | 0 |  | 0 |  |

**Table S3. List of peered at behaviors.** All behaviors and food items that were peered at by individuals for each site. This includes all peering events recorded during 19,000 hours of focal animal sampling at Suaq and 82,000 hours at Tuanan, including all age sex classes (see (Schuppli et al., 2016) for detailed methods). The peered at behavior was always shown by a conspecific (not the peering individual itself). Items: bk= bark, fl= flowers, fr= fruits, ho= honey, ins= insects, lv= leaves, ma= mammal, pi= pith, ro= roots, veg= other vegetative material, wa= water. Numbered techniques are the ones that were also captured by the method of exclusion in (van Schaik et al., 2009) (see table S2). Description of technique/ behavioral element: for feeding, if not specified, the peering was directed at the standard feeding technique of the specific food item. Bold numbers refer to the pictures depicted in figure 4 of the main manuscript.

| **Site** | **Category** | **Species** | **Item** | **Processing steps** | **Description of technique/ behavioral element** |
| --- | --- | --- | --- | --- | --- |
| Suaq | Feeding | Akar asam belimbing | fl | 0 |  |
| Suaq | Feeding | Akar asam belimbing | lv | 0 |  |
| Suaq | Feeding | Akar dedinding | lv | 0 |  |
| Suaq | Feeding | Akar dedinding | veg | 1 |  |
| Suaq | Feeding | Akar jambu koral | lv | 0 |  |
| Suaq | Feeding | Akar jambu koral | pi | 4 | **22** |
| Suaq | Feeding | Akar kantong semar | lv | 0 |  |
| Suaq | Feeding | Akar kekait | lv | 0 |  |
| Suaq | Feeding | Akar kekait | pi | 4 |  |
| Suaq | Feeding | Akar melinjo | veg | 1 |  |
| Suaq | Feeding | Akar pakis | lv | 0 |  |
| Suaq | Feeding | Akar kuning | fr | 2 | **28** |
| Suaq | Feeding | Akar pakis | veg | 1 |  |
| Suaq | Feeding | Akar pakis besar | lv | 0 |  |
| Suaq | Feeding | Akar pakis sarang burung | lv | 1 |  |
| Suaq | Feeding | Akar pakis sarang burung | veg | 1 |  |
| Suaq | Feeding | Akar palo | fr | 1 |  |
| Suaq | Feeding | Akar palo | lv | 0 | **7** |
| Suaq | Feeding | Akar palo | veg | 1 |  |
| Suaq | Feeding | Akar pandang | lv | 0 | **11** |
| Suaq | Feeding | Akar pandang | pi | 2 | **15** |
| Suaq | Feeding | Akar plas | fr | 1 |  |
| Suaq | Feeding | Akar susu kambing | fr | 1 |  |
| Suaq | Feeding | Akar susu kambing | lv | 0 | **9** |
| Suaq | Feeding | Akar susu kambing | veg | 1 | **14** |
| Suaq | Feeding | Akar tikus | lv | 0 |  |
| Suaq | Feeding | Akar tikus | pi | 4 |  |
| Suaq | Feeding | Akar tima tima | lv | 0 |  |
| Suaq | Feeding | Akar tima tima | veg | 1 |  |
| Suaq | Feeding | Akar timun tikus | fr | 3 | **6** |
| Suaq | Feeding | Akar tombang | lv | 0 |  |
| Suaq | Feeding | Akar tombang | pi | 4 | **17** |
| Suaq | Feeding | Akar tombang | veg | 1 |  |
| Suaq | Feeding | Akar tombang besar | veg | 1 |  |
| Suaq | Feeding | Ants | ins | 4 | Ant dead twig length wise parting: Splitting hollow twigs lengthwise and then licking ants out of them. |
| Suaq | Feeding | Ants | ins | 2 | Ant finger dipping: Catching ants from leaves/ branches by touching the leaves with finger tips of the stretched hand and letting them crawls onto the fingers. |
| Suaq | Feeding | Ants | ins | 2 | Ant finger picking: Picking ants off a surface (tree trunk or branches) using the thumb against the side of the index finger. **30** |
| Suaq | Feeding | Ants | ins | 2 | Ant fist fishing: Wiping ants with fist off a surface (tree trunk or branches) using either the side of the fist (along pinkie finger) or front of the fist (along curled fingers). Then licking the (often moving) insects off the hand/ wrist. |
| Suaq | Feeding | Ants | ins | 2 | Ant hollow fist strip: Drawing leaves through the partly closed hand/ mouth to obtain ants that are on the foliage. |
| Suaq | Feeding | Ants | ins | 2 | Ant leaves: Extracting ants with mouth out of leave attachment point. |
| Suaq | Feeding | Ants | ins | 1 | Ant lip licking: Licking insects off a surface (tree trunk, branches) with protruded under lip. |
| Suaq | Feeding | Ants | ins | 1 | Ant lip picking: Picking ants off a surface (tree trunk, branches, leaves, old nest material) with lips (between upper and under lip). |
| Suaq | Feeding | Ants | ins | 3 | Ant nest rip: Extracting ants from an ant nest by ripping open the nest and picking the ants with lips. |
| Suaq | Feeding | Ants | ins | 3 | Ant nest shake: extracting ants out of nest by shaking them out of nest onto hand. |
| Suaq | Feeding | Ants | ins | 2 | c21 Bouquet feeding: using lips to pick ants from fistful of dry, or fresh, or rotting leaves (nests) |
| Suaq | Feeding | Ants | ins | 4 | c23 Dead twig sucking: breaking hollow (dead) twigs to suck ants from inside. **4** |
| Suaq | Feeding | Basong | bk | 2 |  |
| Suaq | Feeding | Basong | fr | 3 |  |
| Suaq | Feeding | Basong | lv | 0 |  |
| Suaq | Feeding | Basong | pi | 4 |  |
| Suaq | Feeding | Bees | ho | 2 | Bee honey licking: licking honey off of bees nest. |
| Suaq | Feeding | Bees | ho | 3 | Bee honey poke: poking fingers into bee nest, then licking fingers off. |
| Suaq | Feeding | Bees | ins | 3 | c15 Branch as swatter: using detached leafy branches to ward off bees/wasps attacking subject (who is raiding their nest) |
| Suaq | Feeding | Bees | ins | 2 |  |
| Suaq | Feeding | Bintangor inai | fr | 0 |  |
| Suaq | Feeding | Caterpillar | ins | 2 | Rolling caterpillars out of leaves. **13** |
| Suaq | Feeding | Caterpillar | ins | 2 |  |
| Suaq | Feeding | Cemenang | bk | 2 |  |
| Suaq | Feeding | Cemenang | fr | 3 |  |
| Suaq | Feeding | Cempedak rawa | fr | 2 |  |
| Suaq | Feeding | Cempedak rawan | fr | 2 |  |
| Suaq | Feeding | Darak gaya | veg | 1 |  |
| Suaq | Feeding | Epiphyte | lv | 0 |  |
| Suaq | Feeding | Gelombang | bk | 2 |  |
| Suaq | Feeding | Gelombang | lv | 0 | **23** |
| Suaq | Feeding | Gersang | bk | 2 |  |
| Suaq | Feeding | Gersang | pi | 4 |  |
| Suaq | Feeding | Jambu air | fr | 1 |  |
| Suaq | Feeding | Kayu geseng | fr | na |  |
| Suaq | Feeding | Kayu kacang | bk | 2 |  |
| Suaq | Feeding | Kayu kacang | fr | 3 |  |
| Suaq | Feeding | Keladih | lv | 0 |  |
| Suaq | Feeding | Keladih | pi | 4 |  |
| Suaq | Feeding | Keladih | veg | 0 |  |
| Suaq | Feeding | Kulibatu | fr | 1 |  |
| Suaq | Feeding | Kulijambu | fl | 0 |  |
| Suaq | Feeding | Kulijambu | fr | 1 |  |
| Suaq | Feeding | Kulijambu | lv | 0 |  |
| Suaq | Feeding | Larvae | ins | 3 | Biting larvae out of Rengas fruits. |
| Suaq | Feeding | Malaka | fr | 3 | Technique for unripe fruits. |
| Suaq | Feeding | Malaka | fr | 3 | Technique for very ripe fruits. |
| Suaq | Feeding | Malaka | fl | 0 |  |
| Suaq | Feeding | Malaka | fr | 2 |  |
| Suaq | Feeding | Mangga hutan | fr | 3 |  |
| Suaq | Feeding | Manggis hutan | fr | 3 |  |
| Suaq | Feeding | Medang baru | fr | 1 |  |
| Suaq | Feeding | Medang baru | lv | 0 |  |
| Suaq | Feeding | Medang baru | pi | 4 |  |
| Suaq | Feeding | Medang burung | fr | na |  |
| Suaq | Feeding | Medang hitam | fr | 3 |  |
| Suaq | Feeding | Medang kersik | fr | 2 |  |
| Suaq | Feeding | Medang lebar daun | fl | 0 |  |
| Suaq | Feeding | Medang lebar daun | lv | 0 |  |
| Suaq | Feeding | Medang nangka | fr | 2 |  |
| Suaq | Feeding | Meranti | bk | 2 |  |
| Suaq | Feeding | Meranti batu | lv | 0 |  |
| Suaq | Feeding | Palem | pi | 2 |  |
| Suaq | Feeding | Palem | veg | 1 |  |
| Suaq | Feeding | Perada | fr | 3 |  |
| Suaq | Feeding | Puin | fr | 2 |  |
| Suaq | Feeding | Puin | lv | 0 |  |
| Suaq | Feeding | Raja penawar | lv | 0 |  |
| Suaq | Feeding | Raja penawar | veg | 0 |  |
| Suaq | Feeding | Raja penawar5 | veg | 0 |  |
| Suaq | Feeding | Raja penawar7 | veg | 0 |  |
| Suaq | Feeding | Raja penawar8 | lv | 0 |  |
| Suaq | Feeding | Rambung | fr | 1 |  |
| Suaq | Feeding | Regah | fr | 3 |  |
| Suaq | Feeding | Regah | veg | 1 |  |
| Suaq | Feeding | Rengas | bk | 2 |  |
| Suaq | Feeding | Rengas | fr | 3 |  |
| Suaq | Feeding | Resak biasa | fr | 1 |  |
| Suaq | Feeding | Resak biasa | lv | 0 |  |
| Suaq | Feeding | Resak payo | fl | 0 |  |
| Suaq | Feeding | Resak payo | fr | 1 |  |
| Suaq | Feeding | Resak payo | lv | 0 | **18** |
| Suaq | Feeding | Resak payo | pi | 4 |  |
| Suaq | Feeding | Resak ubar | fr | 1 |  |
| Suaq | Feeding | Rotan | veg | 1 |  |
| Suaq | Feeding | Rotan tikus | lv | 1 | **24** |
| Suaq | Feeding | Rotan tikus | veg | 1 | **5** |
| Suaq | Feeding | Semangkang | lv | 0 |  |
| Suaq | Feeding | Sepang | fr | 4 | Special technique for very ripe fruits. |
| Suaq | Feeding | Sepang | fl | 0 | **8** |
| Suaq | Feeding | Sepang | fr | 3 |  |
| Suaq | Feeding | Sepang | lv | 0 |  |
| Suaq | Feeding | Sepang | veg | 1 |  |
| Suaq | Feeding | Sitape | lv | 0 |  |
| Suaq | Feeding | Slow loris | ma | 5 | c24 Slow loris eating: capture and eat slow loris hiding in dense vegetation |
| Suaq | Feeding | Squirrel | ma | 5 |  |
| Suaq | Feeding | Sweat bees | ins | 2 | Sweat bees air snatch: Catching flying insects in the air by snatching them with hand. |
| Suaq | Feeding | Sweat bees | ins | 2 | Sweat bees fur smashing: Catching flying insects by smashing them against own fur (mostly upper arm). |
| Suaq | Feeding | Sweat bees | ho | 3 | Sweat bees tree hole poking: Poking sweat bees with fingers out of tree hole. |
| Suaq | Feeding | Sweat bees | ho | 2 | Sweat bees tree hole sucking: Sucking and licking at a tree hole with a sweat bee nest inside to extract honey. **20** |
| Suaq | Feeding | Sweat bees | ins | 2 |  |
| Suaq | Feeding | Tama lava | lv | 0 |  |
| Suaq | Feeding | Tampulicin | fr | 2 |  |
| Suaq | Feeding | Tapis batu | fr | 0 |  |
| Suaq | Feeding | Termites | ins | 2 | Termite dead wood chewing: Eating insects out of dead wood (log or dead tree trunk) by passing (chewing) pieces of dead wood through the mouth. Often the dead wood it bitten/ scratched open before. |
| Suaq | Feeding | Termites | ins | 2 | Termite dead wood sucking: Eating termites out of dead wood (log or dead tree trunk) by sucking them out. Often the dead wood it bitten/ scratched open before. |
| Suaq | Feeding | Termites | ins | 2 | Termite fist fishing: Wiping termites with fist off a surface (tree trunk or branches) using either the side of the fist (along pinkie finger) or front of the fist (along curled fingers). Then licking the (often moving) insects off the hand/ wrist. |
| Suaq | Feeding | Termites | ins | 2 | Termite fist smashing: Smashing insect against surface with front/ side of the fist or the back of the wrist. Then eating the insects off the hand. |
| Suaq | Feeding | Termites | ins | 3 | Termite roots: Extracting termites with mouth from roots of a liana or other vegetation. **16** |
| Suaq | Feeding | Ubar | bk | 2 |  |
| Suaq | Feeding | Ubar | fr | 3 |  |
| Suaq | Feeding | Ubar | lv | 0 |  |
| Suaq | Feeding | Water | wa | 2 | Drinking water from a pitcher plant, by squishing the plant. |
| Suaq | Feeding | Water | wa | 2 | Drinking water from a pitcher plant, using it like a cup. |
| Suaq | Feeding | Water | wa | 1 | Drinking water using hand as a cup. |
| Suaq | Feeding | Water | wa | 1 | Drinking water with mouth from a tree fork. **19** |
| Suaq | Feeding | Water | wa | 1 | Drinking water with mouth from a tree hole. |
| Suaq | Feeding | Water | wa | 2 | i18 Drink from bottom of pitcher plant after biting through bottom (not drink like cup!) |
| Suaq | Moving |  |  |  | Biting through vine to swing Tarzan-style across gap (c36). |
| Suaq | Moving |  |  |  | Brachiating: swinging from tree limb to tree limb by using only arms. **35** |
| Suaq | Nesting |  |  |  | c13 Twig biting: systematically passing ends of twigs used for lining of nest past the mouth (sometimes including actual bite) during last phase of nest building. |
| Suaq | Nesting |  |  |  | c26 Carry leafy branch to different tree in which subject starts to build nest using carried leaves as lining, pillow or cover (Tuanan: Campnosperma). |
| Suaq | Nesting |  |  |  | c6 Bunk nests: build a nest a short distance above the nest used for resting (during rain). |
| Suaq | Nesting |  |  |  | c7 Sun cover: building cover on nest during bright sunshine (rather than rain). **12** |
| Suaq | Nesting |  |  |  | c8 Hide under nest: seek shelter under nest for rain. |
| Suaq | Nesting |  |  |  | Fixing an old nest to then use it for resting. |
| Suaq | Nesting |  |  |  | Making a chair by bending branches into a platform to sit in. **31** |
| Suaq | Nesting |  |  |  | Making a mattress (nest element). |
| Suaq | Nesting |  |  |  | Making a nest using multiple trees. |
| Suaq | Nesting |  |  |  | Making a pillow (nest element). |
| Suaq | Nesting |  |  |  | Making an ordinary nest by bending branches into a platform to then rest in it. **10** |
| Suaq | Other |  |  |  | Auto groom: skin/ hair care of the own body while looking at it. |
| Suaq | Other |  |  |  | Defecating: peering at a conspecific defecating and/ or urinating. |
| Suaq | Other |  |  |  | Exploration: usually destructive manipulation of objects, while intently looking at the object without and with feeding attempt (explorative try feeding). **29** |
| Suaq | Other |  |  |  | Movement play: repetitive, fast movements. |
| Suaq | Other |  |  |  | Nest destruction: taking apart a conspecific's nest. |
| Suaq | Other |  |  |  | Object play: playful manipulation of objects including repetitive movements and no apparent immediate goal (including: c39 (ex-i17) water play (wild splashing) on ground) |
| Suaq | Other |  |  |  | Resting: sitting, lying, standing, hanging while not doing anything else. |
| Suaq | Other |  |  |  | Umbrella use: making an umbrella out of leaves to protect oneself from the rain. **34** |
| Suaq | Social |  |  |  | Agonistic interaction: agonistic action directed at a conspecific (e.g. biting, hitting, pushing). |
| Suaq | Social |  |  |  | Begging: trying to solicit food or any other item from a conspecific. **33** |
| Suaq | Social |  |  |  | c31 Male and female use the same nest to spend the entire night (during consortship). |
| Suaq | Social |  |  |  | c35 Nest as social refuge: female on nest left alone by otherwise coercive male. |
| Suaq | Social |  |  |  | c38(ex-i14) Coercive hand-holding: male firmly holds female’s hand/wrist for long period of time during consort. |
| Suaq | Social |  |  |  | Copulation: A male and a female copulating, intromission achieved. |
| Suaq | Social |  |  |  | Display: displaying at a conspecific by shaking branches and trees. |
| Suaq | Social |  |  |  | Grooming: skin/hair care (with fingers while looking at this) of an other individual. |
| Suaq | Social |  |  |  | i1 GG rub: females rubbing their genitals together. |
| Suaq | Social |  |  |  | Peering/ Social watching: intently watching a conspecific. **26** |
| Suaq | Social |  |  |  | Sex inspection: sniff or touch with mouth or fingers the genital area of a conspecific. |
| Suaq | Social |  |  |  | Social play: playing with a conspecific. **32** |
| Suaq | Social |  |  |  | Social play interspecific: playing with an individual of another species (e.g. siamang or gibbon). **21** |
| Suaq | Social |  |  |  | Sucking: drinking mother's milk (infants). **27** |
| Suaq | Tool |  |  | 5 | Insect swipe tool use: using a long stick tool to swipe off insect off a surface. **2** |
| Suaq | Tool |  |  | 5 | Seed-extraction tool use: using tool to extract seeds from the protected fruits of Neesia sp. (c18). **3** |
| Suaq | Tool |  |  | 5 | Stick as chisel (1) to open termite nest in log or ant nest on ground (i13). |
| Suaq | Tool |  |  | 5 | Tree-hole tool-use: using tool to poke into tree holes to obtain social insects or their products (c17). **1** |
| Suaq | Vocalization |  |  |  | c11 Raspberry: spluttering sounds associated with nest building. |
| Suaq | Vocalization |  |  |  | c2 Kiss squeak with leaves: Using leaves on mouth to amplify sound, then drop leaf. |
| Suaq | Vocalization |  |  |  | c28 Throat scrape: deep throat sound made by female towards offspring. |
| Suaq | Vocalization |  |  |  | Kiss squeak: making a kiss sound like vocalization, mostly in agonistic contexts. **25** |
| Suaq | Vocalization |  |  |  | Long call: Making a long call (flanged males). |
| Tuanan | Feeding | Akar buntut tikus | pi | 0 |  |
| Tuanan | Feeding | Akar buntut tikus | veg |  |  |
| Tuanan | Feeding | Akar dangu | fr | 2 |  |
| Tuanan | Feeding | Akar dangu | fl | 0 |  |
| Tuanan | Feeding | Akar kalamenyu | fr | na |  |
| Tuanan | Feeding | Akar kambalitan | fr | 3 |  |
| Tuanan | Feeding | Akar kamunda | fl | 0 |  |
| Tuanan | Feeding | Akar kamunda | fr | 2 |  |
| Tuanan | Feeding | Akar kamunda | lv | 0 |  |
| Tuanan | Feeding | Akar kecil | fr | na |  |
| Tuanan | Feeding | Akar kecil | lv | 0 |  |
| Tuanan | Feeding | Akar kelakai | ro | 1 |  |
| Tuanan | Feeding | Akar kelanis | lv | 0 |  |
| Tuanan | Feeding | Akar kuning | fr | 2 |  |
| Tuanan | Feeding | Akar tampelas | fr | 1 |  |
| Tuanan | Feeding | Akar uwei nyamei | fr | 0 |  |
| Tuanan | Feeding | Ants | ins | 1 | Ant lip licking: Licking insects off a surface (tree trunk, branches) with protruded under lip. |
| Tuanan | Feeding | Ants | ins | 3 | Ant nest shake: extracting ants out of nest by shaking them out of nest onto hand. |
| Tuanan | Feeding | Ants | ins | 2 | c21 Bouquet feeding: using lips to pick ants from fistful of dry, or fresh, or rotting leaves (nests) |
| Tuanan | Feeding | Behaw punei | veg | na |  |
| Tuanan | Feeding | Bengaris | bk | 2 |  |
| Tuanan | Feeding | Caterpillar | ins | 2 |  |
| Tuanan | Feeding | Enyak beruk | fr | 2 |  |
| Tuanan | Feeding | Epiphyte | veg | 1 |  |
| Tuanan | Feeding | Gerising | fr | 1 |  |
| Tuanan | Feeding | Gerising | pi | 1 |  |
| Tuanan | Feeding | Gerising besar | pi | 1 |  |
| Tuanan | Feeding | Hangkang | fr | 2 |  |
| Tuanan | Feeding | Hanyer bajai | veg | na |  |
| Tuanan | Feeding | Kamuning | lv | 0 |  |
| Tuanan | Feeding | Kapur naga jangkar | lv | 0 |  |
| Tuanan | Feeding | Karandau putih | bk | 2 |  |
| Tuanan | Feeding | Karandau putih | fr | 3 |  |
| Tuanan | Feeding | Katiau | fl | 0 |  |
| Tuanan | Feeding | Katiau | fr | 1 |  |
| Tuanan | Feeding | Keranji | lv | 0 |  |
| Tuanan | Feeding | Kumpang | fr | 3 |  |
| Tuanan | Feeding | Lewang | lv | 0 |  |
| Tuanan | Feeding | Lewang | fr | 2 |  |
| Tuanan | Feeding | Lewang | veg | 1 |  |
| Tuanan | Feeding | Lunuk beringin | lv | 0 |  |
| Tuanan | Feeding | Lunuk besar | fr | 1 |  |
| Tuanan | Feeding | Lunuk besar | veg | 0 |  |
| Tuanan | Feeding | Lunuk handipe | lv | 0 |  |
| Tuanan | Feeding | Lunuk tanah | lv | 0 |  |
| Tuanan | Feeding | Mahandingan | fr | 2 |  |
| Tuanan | Feeding | Mahawai 2 | fr | 2 |  |
| Tuanan | Feeding | Mahawai umb | fr | 2 |  |
| Tuanan | Feeding | Manggis hutan daun besar | fr | 3 |  |
| Tuanan | Feeding | Manggis hutan daun kecil | fr | 3 |  |
| Tuanan | Feeding | Manggis hutan daun kecil | lv | 0 |  |
| Tuanan | Feeding | Mangkinang blawau | fr | 1 |  |
| Tuanan | Feeding | Maruang | bk | 2 |  |
| Tuanan | Feeding | Nyatho undus bua besar | fr | 1 |  |
| Tuanan | Feeding | Nyatho undus bua besar | fl | 0 |  |
| Tuanan | Feeding | Nyatho undus bua merah | fr | 1 |  |
| Tuanan | Feeding | Nyatoh puntik | fl | 2 |  |
| Tuanan | Feeding | Pakan | fr | na |  |
| Tuanan | Feeding | Pantung | bk | 2 |  |
| Tuanan | Feeding | Pantung | fr | 2 |  |
| Tuanan | Feeding | Pantung | pi | 4 |  |
| Tuanan | Feeding | Papung | fr | 2 |  |
| Tuanan | Feeding | Pari pari | fr | 3 |  |
| Tuanan | Feeding | Pendo | fl | 0 |  |
| Tuanan | Feeding | Pendo | lv | 0 |  |
| Tuanan | Feeding | Piais | fr | 2 |  |
| Tuanan | Feeding | Pindin pandan | fr | 3 |  |
| Tuanan | Feeding | Pinding pandan | bk | 2 |  |
| Tuanan | Feeding | Pinding pandan | lv | 0 |  |
| Tuanan | Feeding | Purun tikus | ro | 1 |  |
| Tuanan | Feeding | Rahanjang bawi | fr | 2 |  |
| Tuanan | Feeding | Rahanjang hatue | fr | 2 |  |
| Tuanan | Feeding | Rambutan hutan | fr | 2 |  |
| Tuanan | Feeding | Rengas parei | fr | 0 |  |
| Tuanan | Feeding | Rewui | fr | 1 |  |
| Tuanan | Feeding | Sangkuwuk | ro | 1 |  |
| Tuanan | Feeding | Tagula | fr | 1 |  |
| Tuanan | Feeding | Tampang | fr | 1 |  |
| Tuanan | Feeding | Tantimun | fr | 2 |  |
| Tuanan | Feeding | Tapuhut putih | fr | 1 |  |
| Tuanan | Feeding | Tarantang | fr | 0 |  |
| Tuanan | Feeding | Tatumbu kasar | fr | 2 |  |
| Tuanan | Feeding | Termites | ins | 2 | Termite dead wood chewing: Eating insects out of dead wood (log or dead tree trunk) by passing (chewing) pieces of dead wood through the mouth. Often the dead wood it bitten/ scratched open before. |
| Tuanan | Feeding | Termites | ins | 2 | Termite dead wood sucking: Eating termites out of dead wood (log or dead tree trunk) by sucking them out. Often the dead wood it bitten/ scratched open before. |
| Tuanan | Feeding | Tilap | fr | 1 |  |
| Tuanan | Feeding | Tutup kabali | fr | 3 |  |
| Tuanan | Feeding | Tutup kabali | lv | 0 |  |
| Tuanan | Feeding | Water | wa | 2 | Drinking water from a pitcher plant, using it like a cup. |
| Tuanan | Feeding | Water | wa | 1 | Drinking water from the ground. |
| Tuanan | Feeding | Water | wa | 1 | Drinking water from tree hole. |
| Tuanan | Feeding | Water | wa | 2 | i18 Drink from bottom of pitcher plant after biting through bottom (not drink like cup!) |
| Tuanan | Moving |  |  |  | Moving: goal directed locomotion. |
| Tuanan | Nesting |  |  |  | c13 Twig biting: systematically passing ends of twigs used for lining of nest past the mouth (sometimes including actual bite) during last phase of nest building. |
| Tuanan | Nesting |  |  |  | c26 Carry leafy branch to different tree in which subject starts to build nest using carried leaves as lining, pillow or cover (Tuanan: Campnosperma). |
| Tuanan | Nesting |  |  |  | Fixing an old nest to then use it for resting. |
| Tuanan | Nesting |  |  |  | Making a chair by bending branches into a platform to sit in. |
| Tuanan | Nesting |  |  |  | Making a mattress (nest element). |
| Tuanan | Nesting |  |  |  | Making a nest using multiple trees. |
| Tuanan | Nesting |  |  |  | Making a pillow (nest element). |
| Tuanan | Nesting |  |  |  | Making an ordinary nest by bending branches into a platform to then rest in it. |
| Tuanan | Other |  |  |  | Auto groom: skin/ hair care of the own body while looking at it. |
| Tuanan | Other |  |  |  | c27 Branch cushion: covering (non-spiny) big branch(es) with few leaves or leafy branches, then sit or lie on it. |
| Tuanan | Other |  |  |  | Defecating. |
| Tuanan | Other |  |  |  | Exploration: usually destructive manipulation of objects, while intently looking at the object without and with feeding attempt (explorative try feeding). |
| Tuanan | Other |  |  |  | i24 Washing face and arms with water from tree hole. |
| Tuanan | Other |  |  |  | Movement play: repetitive, fast movements. |
| Tuanan | Other |  |  |  | Object play: playful manipulation of objects including repetitive movements and no apparent immediate goal (including: c39 (ex-i17) Water play (wild splashing) on ground) |
| Tuanan | Other |  |  |  | Wound inspection: inspecting a wound on own body. |
| Tuanan | Social |  |  |  | Begging: trying to solicit food or any other item from a conspecific. |
| Tuanan | Social |  |  |  | c38 (ex-i14) Coercive hand-holding: male firmly holds female’s hand/wrist for long period of time during consort |
| Tuanan | Social |  |  |  | Copulation (including c33 Copulation on female’s nest). |
| Tuanan | Social |  |  |  | Crying. |
| Tuanan | Social |  |  |  | Grooming: skin/hair care (with fingers while looking at this) of another individual. |
| Tuanan | Social |  |  |  | Sex inspection: sniff or touch with mouth or fingers the genital area of a conspecific. |
| Tuanan | Social |  |  |  | Snag crash display: pushing over dead tree trunks, directed at a conspecific. |
| Tuanan | Social |  |  |  | Social play: playing with a conspecific. |
| Tuanan | Social |  |  |  | Suckling: drinking mother's milk (immatures). |
| Tuanan | Vocalization |  |  |  | Bubbling: making bubbling sounds, usually right before starting to long call (flanged males). |
| Tuanan | Vocalization |  |  |  | c2 Kiss squeak with leaves: Using leaves on mouth to amplify sound, then drop leaf. |
| Tuanan | Vocalization |  |  |  | c25 Nest smack: smacking sounds associated with nest building |
| Tuanan | Vocalization |  |  |  | c28 Throat scrape: deep throat sound made by female towards offspring. |
| Tuanan | Vocalization |  |  |  | Kiss squeak: making a kiss sound like vocalization, mostly in agonistic contexts. |

**References supplementary material**

Schuppli, C., Meulman, E., Forss, S. I. F., Aprilinayati, F., Van Noordwijk, M. A., & Van Schaik, C. P. (2016). Observational social learning and socially induced practice of routine skills in wild immature orang-utans. *Animal Behaviour, 119*, 87-98. doi:10.1016/j.anbehav.2016.06.014

van Schaik, C. P., Ancrenaz, M., Djojoasmoro, R., Knott, C. D., Morrogh-Bernard, H. C., Nuzuar, . . . van Noordwijk, M. A. (2009). Orangutan cultures revisited. In S. A. Wich, S. S. Utami Atmoko, T. Mitra Setia, & C. P. van Schaik (Eds.), *Orangutans: geographic variation in behavioral ecology and conservation. [Oxford Biology.]* (pp. 299-309).
